# Doctors’ rural practice self‐efficacy is associated with current and intended small rural locations of practice

**DOI:** 10.1111/ajr.12486

**Published:** 2019-04-07

**Authors:** Megan Bentley, Nadine Dummond, Vivian Isaac, Heidi Hodge, Lucie Walters

**Affiliations:** ^1^ Flinders University Rural Health South Australia Flinders University Mount Gambier South Australia Australia; ^2^ Flinders University Rural Health South Australia Flinders University Renmark South Australia Australia; ^3^ Mid North Knowledge Partnership Flinders University Rural Health South Australia Flinders University Burra South Australia Australia

**Keywords:** medical doctors, rural careers, rural clinical schools, rural practice self‐efficacy

## Abstract

**Objective:**

Key factors which positively influence recruitment and retention of doctors to rural practice include rural background and positive rural training experience. Despite this knowledge, there is no well‐established conceptual framework to explain how these factors influence intention. The aim of this study was to consider rural practice self‐efficacy and its influence on rural career choice by doctors. Questions relating to self‐efficacy were formulated using Bandura's four proposed sources of self‐efficacy, which include mastery experiences, vicarious experience, social persuasion and emotional and physical response to experiences.

**Design:**

Cross‐sectional study.

**Setting and participants:**

Medical school graduates from Flinders University, who completed a rural year as a part of the clinical component of the course between 1997 and 2015.

**Main outcome measures:**

Rural self‐efficacy; current and intended location of practice in small rural communities (<25 000 people).

**Result:**

It was found that 28.5% of participants were currently working in communities of <25 000 people. Levels of intent for future small town rural practice were consistent across career stages after internship. Higher rural practice self‐efficacy scores were found in doctors working in smaller towns (<25 000) and small communities (<10 000). Higher self‐efficacy was also associated with rural background, more senior career status, earlier speciality decision time and smaller expectation‐experience gap.

**Conclusion:**

An independent association exists between rural self‐efficacy and intention to remain or return to small rural practice. The article offers rural clinical schools the opportunity to consider how they can influence future rural career outcomes.

**What is already known on this subject:**

- Medical students who participate in rural clinical school placements have higher rural career outcomes.
- There is argument about whether this finding is due to nature (rural background) or nurture (exposure).
- There is no well‐established conceptual framework to explain how these factors influence intention.

**What this study adds:**

- This cross‐sectional study demonstrates rural practise self‐efficacy is associated with current rural practise and future intention to practise rurally.
- Rural practise self‐efficacy increases with career progression and increases with smaller, more isolated locations of practice.
- Rural practise self‐efficacy offers an explanation of how nature and nurture contribute to rural medical practise intent.

## 1 INTRODUCTION

Recruitment and retention of medical practitioners in rural Australia is an ongoing challenge. In an attempt to address this issue, multiple initiatives have been implemented in recent decades, ranging from bonded medical places to rural financial incentives.^1^ Two consistent factors associated with rural medical practice intention are rural background and positive rural training experiences.^2^, ^3^ Although some studies suggest these factors have additive effects,^4^ not all students take‐up rural careers.^5^ A longitudinal cohort study argues that nature (pre‐existing rural career interest), rather than nurture (during undergraduate rural clinical school [RCS]), affects the likelihood of choosing a rural medical career.^6^ Medical students’ first preference for rural placements and overall wellbeing during these placements correlate with increased rural career interest.^7^, ^8^ Despite new understanding that medical education can transform students’ world view and influence their career trajectories,^9^ there is no well‐established conceptual framework to explain how positive rural experiences influence intention.

Recently, rural practice self‐efficacy has emerged as a concept that might influence rural medical career intent.^10^ Self‐efficacy is defined as “people's beliefs in their capabilities to produce designated levels of performance.”^11^ Four sources of individual self‐efficacy have been proposed: mastery experiences; vicarious learning; social persuasion; and emotional and physical response to experiences.^11^ Rural practice self‐efficacy might provide a meaningful conceptual construct in explaining rural career intention. This study explores the relationship between rural practice self‐efficacy and rural careers in alumni from one Australian RCS.

## 2 METHOD

The survey used in this study included self‐efficacy score, current location of practice, career intent and expectations compared to reality gap. Self‐efficacy was evaluated with five statements using a 5‐point Likert scale (“strongly disagree” = 1 to “strongly agree” = 5). These statements were adapted from Isaac's^10^ Rural Practice Self‐efficacy scale. A total self‐efficacy score was calculated as the sum of the Likert scale scores with reverse scoring for the “too hard” question, giving a total minimum score of 5 and total maximum score of 25.

Current practice location was measured by asking participants to categorise their main location of current practice as one of five options: capital city, major urban centre (>100 000), regional city or large town (>25 000‐100 000), small town (10 000‐25 000) or smaller community (<10 000). A statement of intention to remain in (or return to) small town rural practice (<25 000) in the foreseeable future was scored using a 5‐point Likert scale and recoded, with “strongly disagree” to “neutral” interpreted as no or neutral intent for small rural practice and “agree” or “strongly agree” as positive rural intent.

Expectation‐experience gap was gauged by asking participants to rate their most recent experiences of rural practice in comparison to their memory of their expectations immediately following their full‐year RCS training during medical school across seven domains (from 1 = “substantially less than postRCS expectations” to 5 = “substantially greater than postRCS expectations”). The seven domains include: professional support, personal support, skill level required, breadth of knowledge required, level of autonomy and amenities and opportunities for professional development. Responses of “less,” “somewhat less” or “different” to expectations were coded as expectation‐experience gaps and scored as 1. A total expectation‐experience gap score was then calculated and coded as either: (i) no significant gap, with total score of less than 3; or (ii) expectation‐experience gap, with total score of 3 or more.

The Flinders University Parallel Rural Community Curriculum (PRCC) is a program where medical students spend a full academic year based in general practice participating in a rural, community‐engaged longitudinal integrated clerkship.^12^ In 2016, there were 383 alumni from this program since it commenced in 1997. The 252 alumni with known email addresses were invited by email to participate in an online survey. Data were entered into SPSS (Version 22, IBM SPSS Inc., Armonk, NY, USA). Missing data were excluded from analysis on a variable‐by‐variable basis. Descriptive data were examined to determine the study variables (Table 1). The associations with rural self‐efficacy score were analysed using independent sample *t* test and one‐way ANOVA with a significant *P*‐value <0.05. Multivariate logistic regression was used to calculate odds ratios (ORs) with 95% confidence interval for the association between self‐efficacy and intention to remain or return to rural careers.

**Table 1:** Characteristics of the sample

Characteristics	N	%^a^
Sex		
Female	58	56.9
Male	44	43.1
Rural background		
No	59	57.8
Yes	42	41.6
More than 8 y of rural upbringing		
No	64	62.7
Yes	38	37.3
Partner has rural background		
No	51	50.0
Yes	50	49.0
Current career status		
Completed medical degree	8	7.8
Completed an intern position	6	5.9
Commenced a vocational training program	47	46.1
Completed a vocational training program	41	40.2
Current main location of practice		
Capital city	45	44.1
Major urban centre	14	13.7
Regional city or large town	14	13.7
Smaller town	12	11.8
Small communities	17	16.7
Intent for small rural practice (town <25 000)		
Positive intent	52	50.9
No or neutral intent	50	49.1
Level of agreement with “I am happy with my current location of practice”		
Strongly disagree	4	3.9
Disagree	1	1.0
Neutral	8	7.8
Agree	44	43.1
Strongly agree	43	42.2
Speciality intent or a vocational training commenced or completed		
General practice/GP combination	57	59.9
Other speciality	44	43.1
Speciality decision time		
Prior to PRCC	29	28.4
While studying or after PRCC	19	18.6
Following graduation	44	43.1
Other	10	9.8

PRCC, Parallel Rural Community Curriculum.

a Percentages might not add up to 100% because of missing data.

### 2.1 Ethics approval

Ethics approval was granted by the Flinders University Social and Behavioural Research Ethics Committee (project number 6032).

## 3 RESULTS

The overall response rate was 40.5%, with 102 completed responses received. The majority of the survey respondents were women (n = 58, 56.9%), had commenced or completed vocational training (n = 88, 86.3%), were working outside a capital city (n = 57, 55.9%), were happy with their current location of practice (n = 87, 85.3%), were already in or planning to join general practice (n = 57, 59.9%) and had made their decision regarding speciality training following graduation (n = 44, 43.1%). Of the participant cohort, 29 (28.5%) reported currently practising in towns of <25 000 population. Considering demographics known to be associated with rural practice, 42 (41.6%) reported a rural background, with the majority of these having had >8 years of rural upbringing (n = 38, 37.3%) and 50 (49%) reporting that they had a partner with a rural background.

Small rural practice location increased with career progression, with 25.5% (n = 12) of those who had commenced vocational training and 41.5% (n = 17) of those who had completed training practising in a town of <25 000. Of the participants, 52 (50.9%) expressed positive intent for small rural practice in the future. Levels of positive intent were consistent across all career stages after internship (50%‐55.3%).

**Figure 1:**
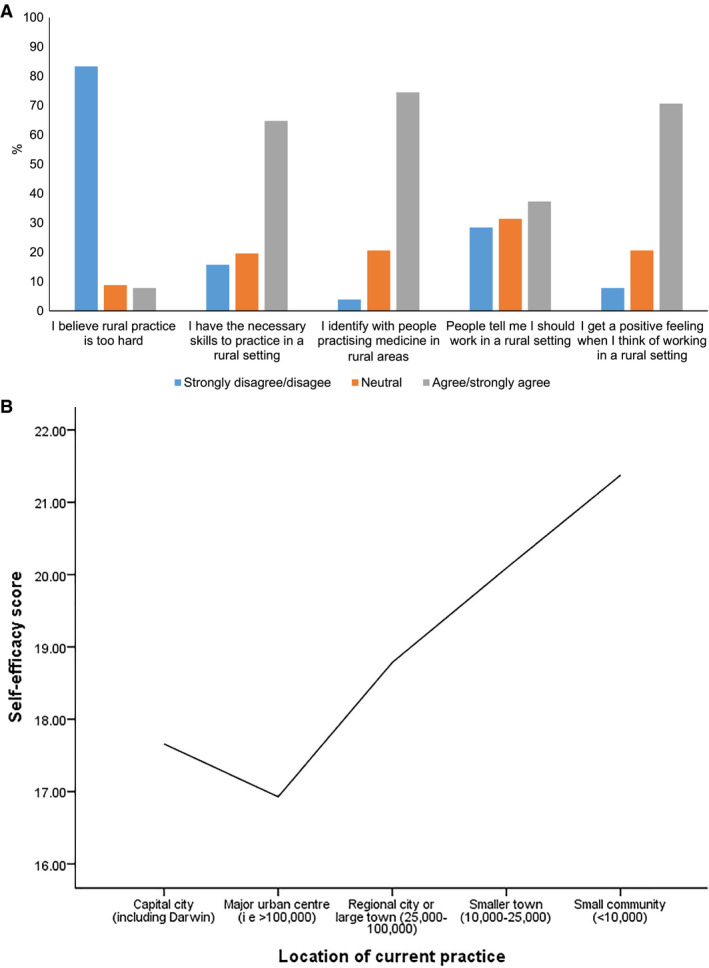
A, Self‐efficacy in rural practice. B, Self‐efficacy score and location of rural practice

Rural practice self‐efficacy questions were answered as outlined in Figure 1A. Mean total score was 18.5 (SD: 3.1) out of a possible 25 with a normal distribution. Mean scores varied with location of practice, with the highest scores found in smaller towns and smaller communities (Figure 1B).

**Figure 2:**
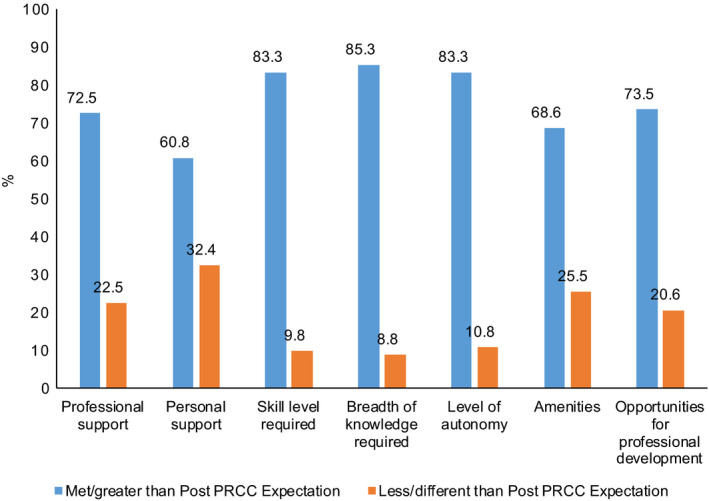
Expectation‐experience gap postrural clinical school. PRCC, Parallel Rural Community Curriculum.

When considering experience compared to expectations, the majority of areas listed were either meeting or exceeding postRCS expectations (Figure 2). Breadth of knowledge required (85.3%), skill level required (83.3%) and extent of autonomy (83.3%) had higher proportions that met or exceeded postRCS expectations. The three areas that had higher proportions that had not met a graduate's postRCS expectations were personal support (32.4%), amenities (25.5%) and professional support (22.5%).

There is a clear positive association between rural practice self‐efficacy and current main location of practice (*f* = 7.2, *P* = 0.001) and future intent to practise in a rural location (*t* = −6.1, *P* = 0.001; Table 2). Higher rural practice self‐efficacy was also significantly associated with rural background (*t* = −2.4, *P* = 0.02), more senior career status (*f* = 3.2, *P* = 0.03), earlier speciality decision time (*f* = 4.6, *P* = 0.012) and lower expectation‐experience gap (*t* = 2.4, *P* = 0.017). There was no significant change in self‐efficacy score with sex, longer upbringing in rural areas, speciality of preference and partner of rural background.

**Table 2:** Associations with rural self‐efficacy

Characteristics	Self‐efficacy score		
	Mean (SD)	*t*/*f*	*P*
Current main location of practice			
Capital city	17.6 (3.1)	7.2	<0.001
Major urban centre	16.9 (2.5)		
Regional city or large town	18.7 (2.8)		
Smaller town	20.1 (2.3)		
Small communities	21.3 (1.9)		
Intention to remain or return to rural practice			
No	16.9 (2.7)	‐6.1	<0.001
Yes	20.2 (2.6)		
Sex			
Female	18.3 (3.4)	‐1.0	0.31
Male	18.9 (2.6)		
Rural background			
No	17.9 (2.6)	‐2.4	0.02
Yes	19.4 (3.5)		
More than 8 y of rural upbringing			
No	18.1 (2.7)	‐1.7	0.08
Yes	19.3 (3.5)		
Partner has rural background			
No	18.3 (2.9)	‐0.8	0.42
Yes	18.8 (3.3)		
Current career status			
Completed medical degree	17.2 (2.3)	3.2	0.03
Completed an intern position	15.6 (2.6)		
Commenced a vocational training program	19.2 (3.0)		
Completed a vocational training program	18.4 (3.0)		
Current main location of practice			
Capital city	17.6 (3.1)	7.2	<0.001
Major urban centre	16.9 (2.5)		
Regional city or large town	18.7 (2.8)		
Smaller town	20.1 (2.3)		
Small communities	21.3 (1.9)		
Speciality intent or a vocational training commenced or completed			
General practice/GP combination	19.0 (3.2)	1.4	0.16
Other speciality	18.1 (2.9)		
Speciality decision time			
Prior to PRCC	20.0 (3.1)	4.6	0.012
While studying or after PRCC	17.9 (2.4)		
Following graduation	17.9 (3.1)		
Expectation‐experience gap			
<3 areas	18.9 (3.0)	2.4	0.025
>3 areas	17.1 (3.0)		

PRCC, Parallel Rural Community Curriculum.

Considering Table 3, multivariate logistic regression for every one point increase in rural practice self‐efficacy, there is a 60% increase in unadjusted intent to remain or return to rural practice (OR, 1.6; 95% CI, 1.3‐1.9; *χ* ^2^ = 21.2; *df* = 1; *P* < 0.001). The association remains consistent once sequentially adjusted for each of the variables associated with rural practice self‐efficacy. For each unit increase in rural practice self‐efficacy, intent to remain or return to rural practice is increased by 50% (OR, 1.5; 95% CI, 1.1‐1.9; *χ* ^2^ = 8.7; *df* = 1; *P* = 0.003).

**Table 3:** Multivariate logistic regression analysis: Independent association between rural self‐efficacy and intention to remain or return to rural practice

	Intention to remain or return to rural practice			
	Individual adjustments		Sequential adjustments	
	OR (95% CI)	*χ* ^2^ (*df*)/*P*	OR (95% CI)	*χ* ^2^ (*df*)/*P*
Unadjusted	1.6 (1.3‐1.9)	21.2 (1)/<0.001	–	–
Sex	1.6 (1.3‐2.0)	21.7 (1)/<0.001	1.6 (1.3‐2.0)	21.7 (1)/<0.001
Rural background	1.6 (1.3‐1.9)	19.8 (1)/<0.001	1.7 (1.3‐2.1)	20.3 (1)/<0.001
Current career status	1.6 (1.3‐2.0)	20.9 (1)/<0.001	1.7 (1.4‐2.1)	20.0 (1)/<0.001
Current location of practice	1.4 (1.2‐1.7)	13.2 (1)/<0.001	1.6 (1.2‐2.0)	12.9 (1)/<0.001
Speciality decision time	1.5 (1.2‐1.8)	15.5 (1)/<0.001	1.5 (1.2‐2.0)	11.4 (1)/0.001
Experience‐expectation gap	1.5 (1.2‐1.8)	17.0 (1)/<0.001	1.5 (1.1‐1.9)	8.7 (1)/0.003

CI, confidence interval; OR, odds ratio.

## 4 DISCUSSION

Previous studies have demonstrated that self‐efficacy is associated with increased rural career intent among students following time spent on rural student placements.^10^, ^13^ This study extends these findings to demonstrate a strong association between rural practice self‐efficacy and current and intended rural location of practice among qualified doctors who participated in a year‐long rural immersion programme during their medical course. We also demonstrated that doctors from smaller towns and communities exhibited higher rural practice self‐efficacy scores, compared with regional and metropolitan areas. The authors purport the clinical experience gained from working in rural areas fosters development of rural self‐efficacy beliefs among these individuals. Importantly, rural self‐efficacy remains strongly positively associated with intention to remain in or return to small rural practice, independent of sex, rural background, current career status, current location of practice, speciality decision time or experience‐expectation gap.

The study findings indicate that more experienced medical graduates have increased rural practice self‐efficacy compared with their colleagues in training. The effect of experience cannot be confirmed, however, as this survey provides a snapshot in time, rather than longitudinal tracking. Maturation of general practice self‐efficacy has been demonstrated previously among registrars.^14^ Given the growth of knowledge and skills in rural practice over time, experience and meaningful feedback are likely the causative factors in developing rural practice self‐efficacy.

Interestingly, this study finds no significant change in self‐efficacy with speciality preference, suggesting that rural practice self‐efficacy is related to the context of practice and might be generalisable across specialities other than general practice. With many currently practising rural doctors having an urban background, measuring and influencing rural practice self‐efficacy in urban background medical students and junior doctors might have a significant rural workforce outcome. The potential for rural clinical placements to influence rural practice self‐efficacy is timely to consider as the Australian Government rolls out Regional Training Hubs^15^ and Rural Junior Doctor Innovation Funding^16^ to connect medical school, prevocational and vocational training in rural areas across Australia.

A salient feature of this study is that we considered whether mismatch between expectations and experience was an independent variable or part of the same story as self‐efficacy. We found that higher rural practice self‐efficacy was significantly associated with a small expectation‐experience gap (when there were <3 domains from those listed in our survey demonstrating a gap). It was heartening to observe that the domains listed were either meeting or exceeding postRCS expectations for the majority of participants, with the most commonly met being breadth of knowledge required, extent of autonomy and required skill level. Our study showed that the three domains which most frequently did not meet a graduate's postRCS expectations were personal support, amenities and professional support. These provide a starting point for considering rural medical workforce recruitment and retention initiatives. In addition, if expectations are too high, it is possible that these negatively affect rural career intent in our study, unmet expectations negatively impacted self‐efficacy. This demonstrates the responsibility RCSs have in ensuring students develop realistic expectations of rural practice while on placement.

The main limitation of this study is that it is a small, single university project and therefore caution needs to be used in applying these results to graduates of other Australian institutions. The study participants included only medical graduates who had completed the longitudinal integrated immersion program, known as the Flinders University PRCC, between 1997 and 2015. The sample demographics, with 56.9% women and 41.6% rural background, are similar to those reported in RCSs across Australia.^4^, ^10^ Interest in the speciality of general practice or rural medicine of 59.9% is consistent with previously reported Flinders University alumni^17^; however, it is significantly higher than national RCS intention data that hover around 29% and other RCS graduate studies of around 24%.^2^, ^10^ Further research is required to explore the impact of general practice‐based RCS placements on rural workforce outcomes. A larger and more diverse sample across multiple universities and across graduate and undergraduate medical degrees would increase the generalisability of findings.

## 5 CONCLUSION

From the current study we have shown that as rural practice self‐efficacy increases, so too does the small town rural practice intent of the individual. Therefore, we propose that fostering the development of an individual's rural practice self‐efficacy beliefs might contribute to recruitment and retention of rural GPs within small rural communities in Australia.

## AUTHORS CONTRIBUTION

MB, ND, HH and LW made substantial contributions to the conception or design of the work; MB and ND led the acquisition of data and wrote the first draft of the paper; VI and LW led the analysis of data and revised critically the first draft for important intellectual content. All authors contributed to the interpretation of data and to final approval of the version to be published, and agree to be accountable for all aspects of the work in ensuring that questions related to the accuracy or integrity of any part of the work are appropriately investigated and resolved.

## DISCLOSURE

Flinders Rural Health South Australia is funded through the Australian Government Rural Health and Medical Training program.
